# Retraction: Assessment of Serum Uric Acid Levels Among Gestational Diabetes Patients in Erbil City

**DOI:** 10.7759/cureus.r205

**Published:** 2025-11-20

**Authors:** Lezma G Qadir, Jwan M Zangana

**Affiliations:** 1 Family Medicine, Kurdistan Higher Council of Medical Specialties, Erbil, IRQ; 2 Family Medicine, College of Medicine, Hawler Medical University, Erbil, IRQ

The Editors-in-Chief have retracted this article due to implausible and exaggerated statistical results, limited multivariable adjustment, and the absence of standardized diagnostic methodology for gestational diabetes mellitus (GDM), which render the findings unreliable. Although metadata was embedded within the SPSS file, it was not provided in a separate codebook, and the raw data supplied was insufficient to validate or replicate the reported analyses. As a result, the study’s conclusions cannot be considered credible. The authors agree with the decision to retract.

